# The clinical significance of microRNA-409 in pancreatic carcinoma and associated tumor cellular functions

**DOI:** 10.1080/21655979.2021.1956404

**Published:** 2021-08-02

**Authors:** Kui Long, Qingbin Zeng, Wenzhi Dong

**Affiliations:** Department of Three Wards of Hepatobiliary Surgery, The Second Affiliated Hospital of Kunming Medical University, Kunming, Yunnan, China

**Keywords:** miR-409, pancreatic carcinoma, clinical significance, cellular functions

## Abstract

In recent years, the increasing incidence of pancreatic carcinoma (PC) patients has become one of the hot issues in the world. microRNAs (miRNAs) can act as oncogenes or tumor suppressor genes and have unpredictable effects on tumors, thus affecting the prognosis and survival of cancer patients. In this paper, we mainly studied the role of microRNA (miR)-409 in PC. The expression levels of miR-409 were analyzed by qRT-PCR. Kaplan-Meier curve and Cox regression were used to analyze the relationship between miR-409 and patient prognosis. The effects of miR-409 on the abilities of proliferation, migration and invasion were detected by CCK-8 and Transwell. The expression levels of miR-409 were down-regulated in PC, compared with normal controls. The prognosis of patients with low miR-409 expression is significantly poor in comparison with those with high expression. The down-regulation of miR-409 was conducive to the proliferation, migration and invasion of PC cells. miR-409 is a tumor suppressor of PC, the clinical significance of miR-409 in pancreatic cancer and related tumor cell function was clarified.

## Graphical Abstract

**Figure:**
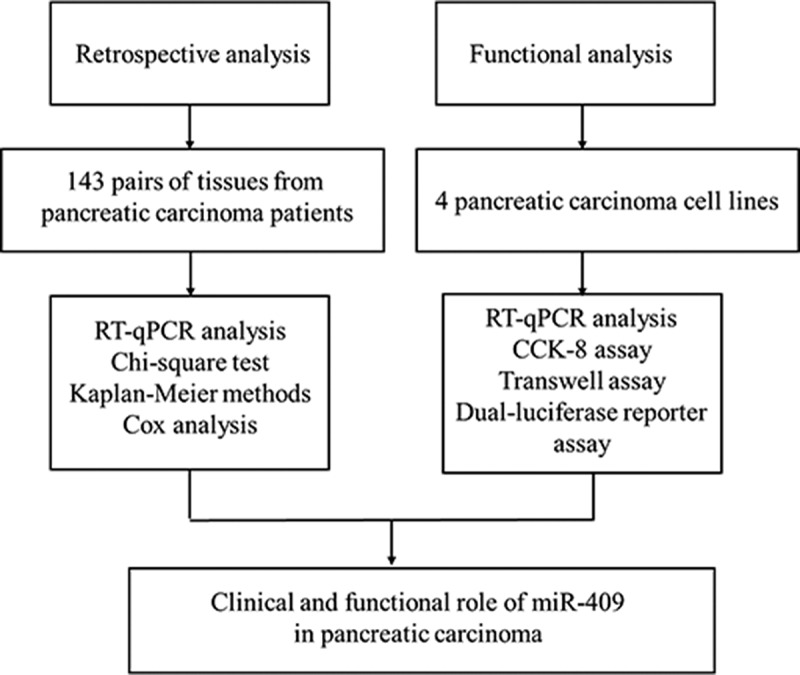

## Introduction

Pancreatic carcinoma (PC) is a common malignant tumor of the digestive tract [1,2]. Its incidence rate and mortality rate are increasing year by year, and it has become one of the main reasons that endanger human life and health [3]. Because of the lack of typical clinical manifestations in the early stage of PC, the tumor shows progressive growth and early metastasis, which makes the majority of patients clinically diagnosed with PC often in the middle and advanced stage, lost the best opportunity for treatment, and the 5-year survival rate is also low [4]. The situation of diagnosis and treatment of PC is grim. Improving the prognosis of pancreatic cancer has been one of the important topics for clinicians. The exploration of specific tumor markers as prognostic biomarkers of PC has become a research hotspot in recent years [2,5].

microRNAs (miRNAs) usually consist of 19–25 nucleotides, with an average of 22 nucleotides [6,7]. These miRNAs usually target one or more mRNAs, which cause degradation or translation inhibition of target mRNA by pairing with specific bases of target mRNA and regulate gene expression at the post-transcriptional level, thus regulating cell differentiation, growth, proliferation, metabolism, apoptosis, and other functions [8–10]. In recent years, studies have revealed that many miRNAs are involved in the biological regulation of cancer, indirectly playing the role of tumor suppressor genes and oncogenes [11,12]. In the studies about PC, many abnormally expressed miRNAs are involved in the tumor progression and have clinical significance, such as miR-132 [13] and miR-27a-3p [14]. The expression of serum miR-409 was found to be decreased in PC and suggesting that miR-409 could be a potential biomarker for the diagnosis of PC [15]. Whereas, the expression of miR-409 in tumor tissues and its clinical and functional role remain elusive in PC.

miR-409 may have a crucial clinical or functional role in PC. This study aimed to investigate the prognostic value and clinical significance of miR-409 in PC using the patients’ tumor tissues. The expression levels of miR-409 in cell tissues and cell lines of PC patients were measured. Through the collected clinicopathological data, the relationship between miR-409 and the patient prognosis was also analyzed. Meanwhile, we further studied the proliferation, migration, and invasion abilities of cells, proving the important role of miR-409 in PC.

## Materials and methods

### Patients and tissue samples

A total of 143 pairs of PC tissues and adjacent normal tissues from patients were collected by surgery in the Second Affiliated Hospital of Kunming Medical University during October 2011 and October 2015. All the tissue samples collected were immediately frozen in liquid nitrogen and restored at −80°C until use. There were no patients who had received other adjuvant treatment including chemotherapy or radiotherapy before surgery and all samples were histopathologically confirmed. The clinical characteristics of PC patients were recorded in detail, including gender, age, tumor size, TNM stage, and lymph node metastasis. All patients signed the informed consent form, and the experiment was supported by the Medical Ethics Committee of The Second Affiliated Hospital of Kunming Medical University. The time of the initial operation was recorded and the patients were followed up regularly for 5 years.

### Cell culture and transfection

The human PC cell lines, PANC-1 and SW1990, BxPC3 and AsPC-1 were purchased from the American type culture collection, the human pancreatic ductal epithelial cell line (HPDE) was purchased from the Cell Repository, Chinese Academy of Sciences (Shanghai, China). All cell lines were routinely cultured in RPMI 1640 medium (11,875,119; HyClone, USA) and 10% fetal bovine serum (FBS; 10,099; Thermo Fisher Scientific, Waltham, USA) were supplemented into the mixture. All cells were cultured at 37°C with 5% CO_2_.

Six-well plates were used for cell transfection. Based on the mature sequences of miR-409, miR-409 mimics (5ʹ- GAAUGUUGCUCGGUGAACCCCU-3ʹ), miR-409 inhibitors (5ʹ-AGGGGUUCACCGAGCAACAUUC-3ʹ), and corresponding negative controls (NCs; mimic NC, 5′-UUCUCCGAACGUGUCACGUTT-3′, inhibitor NC, 5′-CAGUACUUUUGUGUAGUACAA-3′) were purchased from RiboBio, Co., Ltd. (Guangzhou, China) and transfected into the cells according to the manufacturer’s using Lipofectamine 2000 (11,668,019; Thermo Fisher Scientific, Waltham, MA, USA).

### RNA extraction and quantitative real-time PCR

Total RNA was extracted from PC tissue using TRIzol reagent (15,596,018; Thermo Fisher Scientific, Waltham, MA, USA). The concentration and purity of RNA were measured by NanoDrop 2000 (Thermo Fisher Scientific, Waltham, USA). Then, miRNA was reverse transcribed to cDNA using the TaqMan miRNA reverse transcription kit (4,366,597; Thermo Fisher Scientific). The following Real-time quantitative PCR (qRT-PCR) were carried out in triplicate by biosystems 7500 apparatus and SYBR-Green I Master mix kit (Invitrogen; Thermo Fisher Scientific, Waltham, USA) was used to evaluate the amount of PCR products. miR-409 levels were calculated by the 2^−ΔΔCt^ method by normalized to U6 snRNA. In this experiments, Reaction conditions were 95°C for 4 min, followed by 35 cycles of 94°C for 30 sec, 58°C for 20 sec, and 72°C for 20 sec. Primer sequences were miR-409 forward: 5ʹ-GCCGAGAGGGGTTCACCGAG-3ʹ and reverse: 5ʹ-GCAGGGTCCGAGGTATTC-3ʹ; and U6 forward: 5′-CTCGCTTCGGCAGCACA-3′; and reverse: 5′-AACGCTTCACGAATTTGCGT-3′.

### Cell proliferation assay

The Cell Counting Kit-8 (CK04; CCK-8, Dojindo Laboratories, Kumamoto, Japan) [16] was used to assess the ability of cell proliferation. Briefly, the transfected SW1990 and BxPC3 cells (2 × 10^3^ cells/well) were seeded into 96-well plates and cultured overnight. Cells were treated with CCK-8 reagent at 0, 24, 48, and 72 hours. The absorbance was measured at 450 nm, and the value represented the proliferation capacity of CCK-8. All experiments were repeated three times.

### Cell migration and invasion assays

The Transwell chambers used in the cell migration and invasion experiments [17] were 8-μm pore size. For cell migration, 3 × 10^5^ cells in 200 μL of serum-free medium were placed in the upper chamber and the upper chamber was not coated with Matrigel (BD, USA). The lower chamber was filled with 500 μl medium containing 10% FBS. Then, 4% paraformaldehyde solution was used to fix the cells, 0.1% crystal violet was used to stain the cells.

The difference between the cell invasion experiment and the migration experiment was that Matrigel was precoated on the bottom of the upper chamber before the cell invasion experiment. The other steps were the same.

### Bioinformatic analysis

Three online bioinformatics databases, including TargetScan (www.targetscan.org), miRDB (mirdb.org), and CancerMIRNome (http://bioinfo.jialab-ucr.org/CancerMIRNome/) were used to explore the potential target genes and the binding sites of miR-409. The potential target genes were filtered from the three databases using the Venny tool (Venny 2.1.0) and listed in Supplementary Table 1.

**Table 1.** Association between miR-409 expression and different clinical characteristics of patients with pancreatic carcinoma

Clinical characteristics	Cases	miR-409 expression		*P* values
	n = 143	Low (n = 72)	High (n = 71)	
Age				0.677
≤ 55	70	34	36	
> 55	73	38	35	
Gender				0.454
Male	79	42	37	
Female	64	30	34	
Location				0.450
Head of pancreas	89	47	42	
Others	54	25	29	
CEA (μg/ml)				0.681
< 4.3	81	42	39	
≥ 4.3	62	30	32	
CA199 (U/mL)				0.649
< 37	90	44	46	
≥ 37	53	28	25	
Tumor size (cm)				0.357
< 2	70	38	32	
≥ 2	73	34	39	
Differentiation				0.678
Well-moderate	68	33	35	
Poor	75	39	36	
Lymph node metastasis				0.001
Negative	96	39	57	
Positive	47	33	14	
TNM stage				0.004
I-II	108	47	61	
III	35	25	10	

### Dual-luciferase reporter assay

The wild-type or mutant 3ʹ-UTR of GAB1 was amplified and cloned into a pmirGLO reporter vector (Promega, Madison, USA) to construct the GAB1 3ʹ-UTR WT or GAB1 3ʹ-UTR MUT vectors. SW1990 and BxPC3 cells were seeded in 96-well plates and cultured for 24 h. Then cells were transfected with GAB1 3ʹ-UTR WT or GAB1 3ʹ-UTR MUT and miR-409 mimics, inhibitors, or NCs using Lipofectamine 2000 (11,668,019; Thermo Fisher Scientific) for 24 h. Then, a dual-luciferase reporter assay system (Promega, Madison, WI, USA) was used to measure the fire luciferase activities that were normalized to *Renilla* luciferase activities.

### Statistical analysis

Statistical analyses were performed with SPSS version 22.0 software (IBM, Armonk, NY, USA) and GraphPad Prism 5.0 (GraphPad Software, Inc., La Jolla, CA, USA). Student’s t-test and one-way analysis of variance (ANOVA) test were used to test statistical significance. All results were expressed as the mean ± standard deviation (SD). The patient prognostic value was estimated by the Kaplan-Meier curve. Factors related to the overall survival were assessed using Cox regression. It was considered statistically significant when the value of P was less than 0.05.

## Results

The study was carried out to explore the clinical significance and functional role of miR-409 in PC. RT-PCR was used to detect the expression levels of miR-409 in PC tissues and cells. Chi-square test was used to analyze the association between miR-409 expression and clinical parameters of patients, Kaplan-Meier method and Cox regression analysis were performed to explore the clinical role of miR-409 in PC. CCK-8 assay, Transwell assay, and Dual-luciferase reporter assay were carried out to assess the functional role of miR-409 in PC cells.

### Expression of miR-409 in PC tissues and cell lines

**Figure 1.**
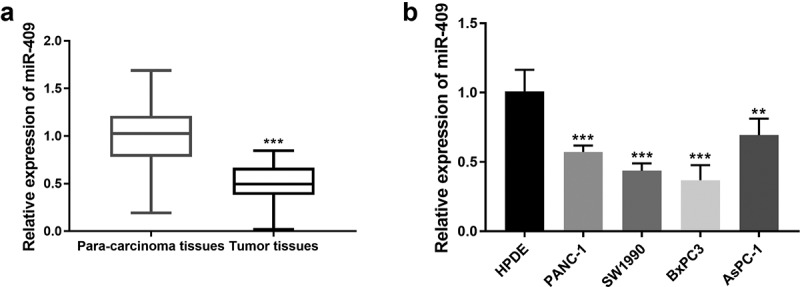
The expression of miR-409 was reduced in PC tissues and cell lines. (a) The expression levels of miR-409 in PC tissue samples and para-carcinoma tissues. ***P < 0.001 (b) The expression levels of miR-409 in 4 PC cell lines. **P < 0.01, ***P < 0.001

The clinical characteristics of 143 PC patients were recorded and qRT-PCR were used to detect the expression level of miR-409 in PC tissue samples and adjacent normal tissues. As shown in Figure 1a, the expression of miR-409 was down-regulated in PC tissues compared with adjacent normal tissues (*P* < 0.001). miR-409 expression in PC cell lines (PANC-1 and SW1990, BxPC3 and AsPC-1) was also significantly lower than that in normal cell lines (HPDE) (*P* < 0.001, Figure 1b).

### Relationship between clinicopathological characteristics and miR-409 expression in PC patients

To facilitate follow-up studies, 143 PC Patients were divided into two groups by the median miR-409 expression, including 71 cases in the high expression group and 72 cases in the low expression group. Table 1 showed the relationship between miR-409 expression and patients’ different clinical characteristics. The results revealed that the expression level of miR-409 was closely associated with lymph node metastasis (*P* = 0.001) and TNM staging (*P* = 0.004). But, the expression of miR-409 was not significantly correlated with age, gender, location, carcinoma embryonic antigen (CEA), CA199, tumor size and differentiation (*P* > 0.05).

### Downregulation of miR-409 is associated with poor prognosis in PC patients

**Figure 2.**
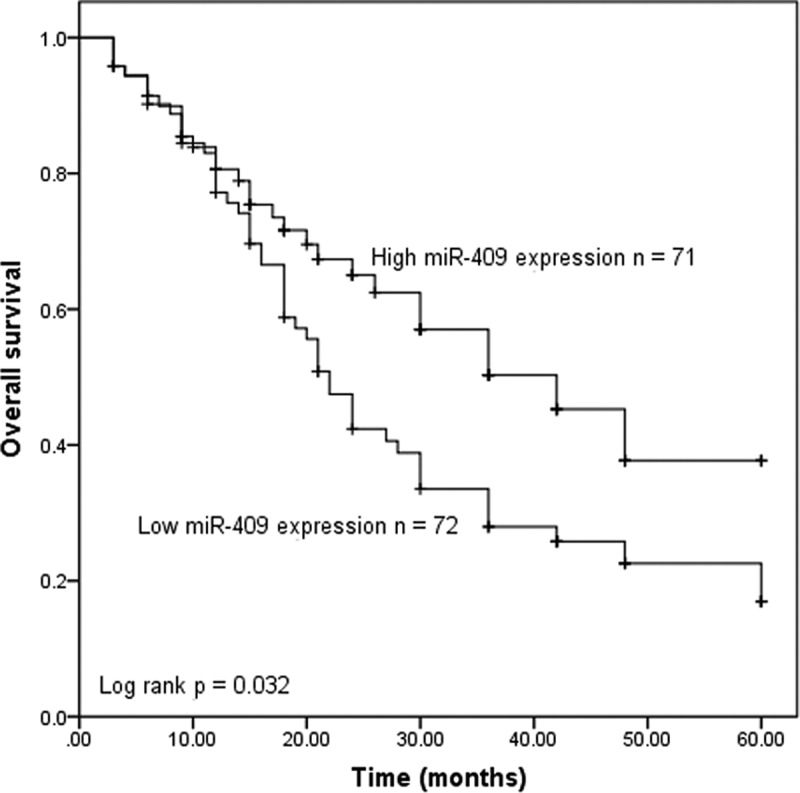
Kaplan-Meier survival curve in relation to the miR-409 expression level in patients with PC. (log-rank test *P* = 0.032)

Kaplan-Meier survival curve and log-rank test were used to evaluate whether miR-409 expression in tumor samples had the prognostic potential for overall survival. As shown in Figure 2, the overall survival of the patients with the low miR-409 expression group was significantly shorter, compared with the patients with high miR-409 expression (*P* = 0.032). To obtain the independent factors affecting the patients’ prognosis, the multivariate Cox regression analysis on the 5-year survival was performed. The results confirmed that miR-409 expression (hazard ratio [HR] = 2.385, *P* = 0.001), lymph node metastasis (HR = 1.798, *P* = 0.030) and TNM stage (HR = 1.870, *P* = 0.035) were the independent prognostic indicators of PC patients (shown in Table 2).

**Table 2.** Multivariate Cox analysis of factors for survival of pancreatic carcinoma patients

Variables	Multivariate Cox analysis		
	HR	95%CI	*P* value
miR-409	2.385	1.404–4.050	0.001
Age	1.027	0.637–1.656	0.913
Gender	0.915	0.562–1.489	0.721
Location	0.893	0.551–1.447	0.646
CEA	0.903	0.563–1.448	0.671
CA199	1.394	0.844–2.303	0.195
Tumor size	0.687	0.429–1.099	0.117
Differentiation	0.954	0.603–1.512	0.843
Lymph node metastasis	1.798	1.060–3.051	0.030
TNM stage	1.870	1.044–3.352	0.035

### Downregulation of miR-409 promotes PC cell proliferation, migration, and invasion

**Figure 3.**
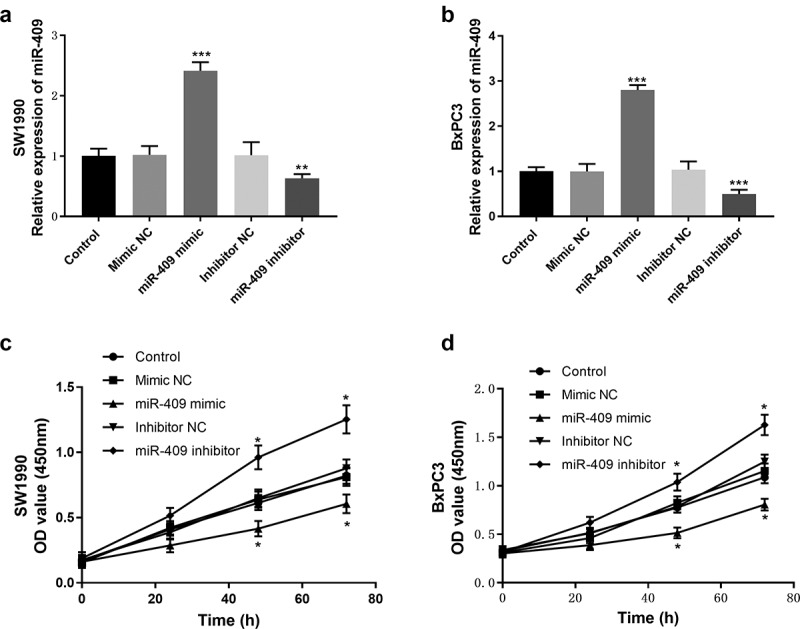
Effects of miR-409 expression levels on proliferation in SW1990 and BxPC3 cells. (a and b) the expression level of miR-409 was analyzed by qRT-PCR after transient transfection with miR-409 mimic/inhibitor (or mimic/inhibitor NC). (c and d) The CCK-8 assay was performed to study cell proliferation. *P < 0.05, **P < 0.01, ***P < 0.001

Cell proliferation, migration, and invasion are crucial to tumor progression, so it is necessary to investigate the effect of miR-409 expression level on tumor cellular functions. The expression levels of miR-409 in SW1990 and BxPC-3 cell lines were lower than those of the other two PC cell lines. Therefore, SW1990 and BxPC-3 cell lines were selected as the research objects for tumor cellular functions experiments. qRT-PCR was used to detect the expression levels of miR-409 after cell transfection. The results indicated that miR-409 mimics could increase the expression levels of miR-409, while miR-409 inhibitors could decrease its expression levels, compared with mimic/inhibitor NC (*P* < 0.01, Figure 3a). Then, the cell proliferation ability was analyzed using the CCK-8 assay. Overexpression of miR-409 inhibited the proliferation of PC cells, while down-regulation of miR-409 had the opposite effect, compared with the control group (Figure 3b, *p* < 0.05).

**Figure 4.**
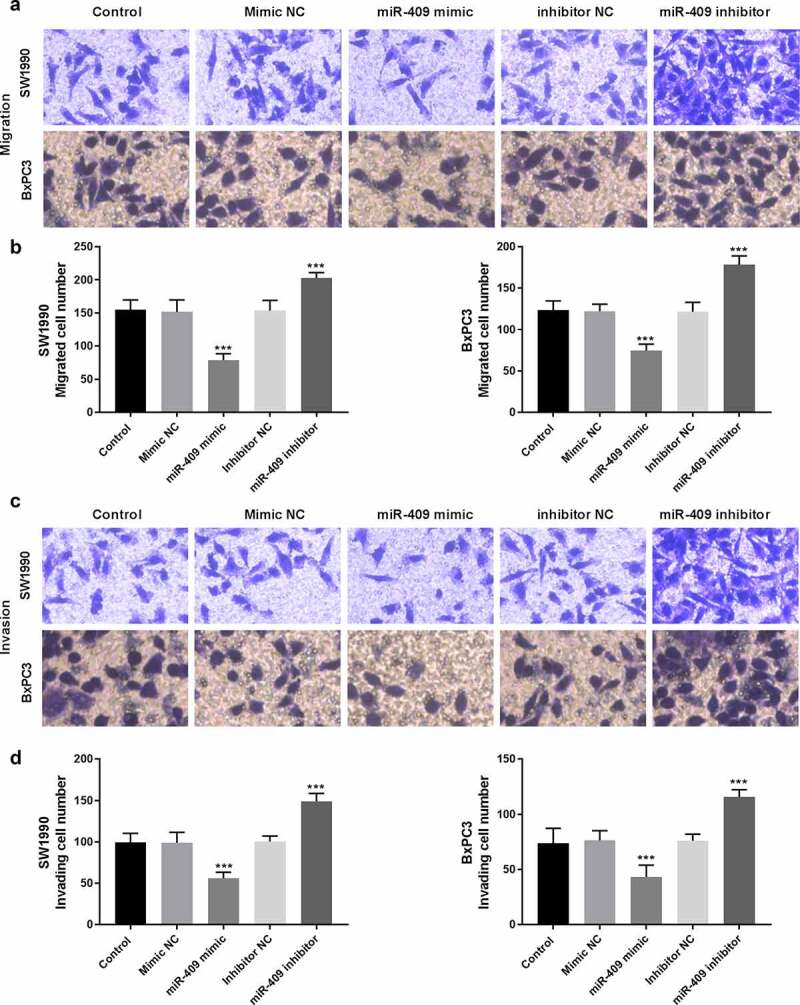
Effects of miR-409 on cell migration and invasion abilities in SW1990 and BxPC3 cells. (a) Presentative pictures of transwell migration assay (200 × magnification). (b) Effects of miR-409 on cell migration. (c) Presentative pictures of transwell invasion assay (200 × magnification). (d) Effects of miR-409 on invasion abilities. ***P < 0.001

Transwell assays were commonly used to analyze the migratory and invasive abilities of tumor cells. The results showed that miR-409 mimic significantly inhibited the invasion and migration of PC cells, while miR-409 inhibitor had a promoting effect, compared with the control group (*P* < 0.001, Figure 4a-Figure 4d).

### GAB1 is a direct target of miR-409 in PC

**Figure 5.**
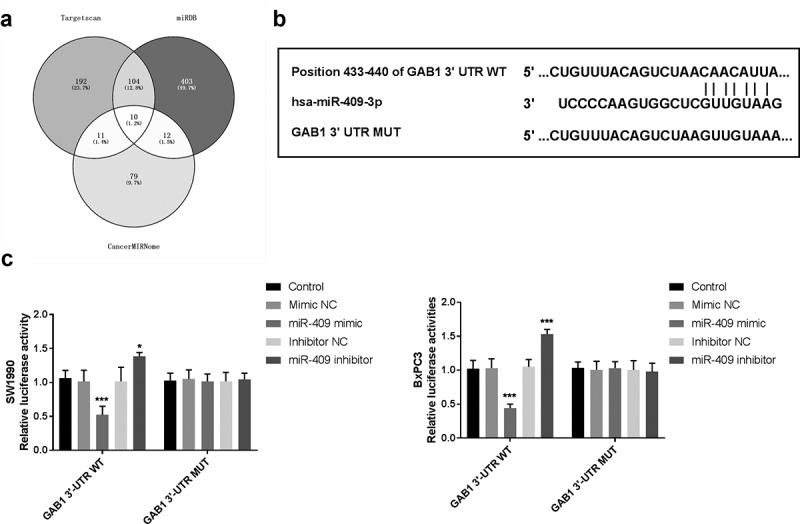
GAB1 may be a direct target of miR-409. (a) Venny tool filtered the potential target of miR-409 from Targetscan, miRDB, and CancerMIRNome online databases. (b) The predicted binding sites between GAB1 3ʹ-UTR and miR-409. (c) Luciferase activity was detected in SW1990 and BxPC3 cells cotransfected with miR-409 mimic or miR-NC and GAB1-3′-UTR WT and GAB1-3′-UTR MUT. *P < 0.05, ***P < 0.001

To explore the molecular mechanisms underlying the function of miR-409 in PC, bioinformatics analysis and dual-luciferase reporter assay were used to predict the putative targets of miR-409. Among these targets that were predicted using bioinformatic analysis (Figure 5a), GAB1 was reported to play an oncogenic role in PC cells through regulating cell proliferation and apoptosis [18]. The predicted binding sites of GAB1 3ʹ-UTR and miR-409 were listed in Figure 5b. Luciferase reporter assay was used to further verify the predicted interactions between GAB1 and miR-409. The results in Figure 5c manifested increased expression of miR-409 by miR-409 mimic weakened luciferase activity in GAB1 3ʹ-UTR WT group, while in GAB1 3ʹ-UTR MUT groups, this inhibition was abolished (*P* < 0.05).

## Discussion

Studies have shown that miRNAs are inextricably associated with tumors. Different types of cancer have different miRNA expression profiles, which can distinguish adjacent normal tissues [19–21]. The discovery of miRNA not only provides new insights into the regulation mechanism of cell proliferation, differentiation, apoptosis but also promotes the development of diagnosis and treatment of diseases [22,23]. The biological characteristics of PC are closely related to the up-regulation and down-regulation of miRNA expression in PC [11,24]. Up-regulation or down-regulation of miRNA expression of the target can cause changes in the expression of the target protein, which plays a role similar to oncogene and tumor suppressor gene, which provides the possibility for the early diagnosis, molecular mechanism and gene therapy of PC [2].

miR-409, located on the chromosome region 14q32, has been found to play a tumor suppressor role in a variety of tumors [25,26]. In the present, miR-409 was abnormally low expressed in PC tissues and cells, which was consistent with previous research data in the study of Kwondo Kim, miR-409 was also downregulated in the genome-wide expression analysis of serum miRNAs in PC patients [15]. In osteosarcoma, the expression levels of miR-409 were reduced in osteosarcoma tissues and cells and revealed a poor prognosis, miR-409 could act as a potential tumor suppressor for osteosarcoma [25]. The occurrence and progression of the tumor are closely related to the abnormal expression of miRNA in patients. Therefore, we further studied the clinical significance of miR-409 for PC and its guiding role in the prognosis of patients.

Analyzing the clinical characteristics of PC patients, low miR-409 expression is closely related to the high TNM stage and positive lymph node metastasis. The lymph node metastasis and TNM stage are important prognostic indicators of tumor patients. Besides, patients with low miR-409 expression have also been shown to have a poor prognosis. Based on the above studies, miR-409 may have a prognosis predictor value of PC patients. Similar results have been reported for other cancers. For example, in breast cancer, the decreased level of miR-409 expression is related to the prognosis of patients, which may be a promising predictor of breast cancer recurrence [27].

Proliferation, migration and invasion of tumor cells are important processes in the development of cancer [28,29]. For instance, miR-409-3p may delay the proliferation of tongue squamous cell carcinoma cells by inhibiting of radixin (RDX) to decrease its migratory and invasive abilities, which may be a potential target for the clinical treatment of tongue squamous cell carcinoma [30]. In addition, miR-409 was down-regulated in bladder cancer, and miR-409 inhibited migration and invasion of bladder cancer cells via targeting c-Met [31]. Besides, miR-409 could function as a tumor suppressor in several cancers by targeting zinc-finger E-box-binding homeobox-1 (ZEB1) [25], E74-like factor 2 (ELF2) [26], and GRB2-associated binding protein 1 (GAB1) [32]. In the present study, dual-luciferase reporter assay revealed that GAB1 was a direct target of miR-409 in PC cells. A previous study has demonstrated that downregulation of GAB1 could inhibit cell proliferation and induced apoptosis of PC cells (PANC-1) [18]. Thus, it is speculated that miR-409 may restain the progression of PC by targeting GAB1. Although we have studied the prognostic significance of miR-409 for PC on the existing basis, it is a pity that a large enough sample size was not selected in the experiment, and the detailed mechanism of miR-409 was not studied in depth. Further studies will be carried out in the subsequent work.

## Conclusion

In conclusion, the expression of miR-409 was down-regulated in PC. PC patients with low miR-409 expression usually have a poor prognosis. The low miR-409 expression may accelerate the tumor cell progression of PC by targeting GAB1. miR-409 may have important clinical significance for the prognosis of patients with PC, providing new ideas and bringing new hope for the treatment of PC.

## Supplementary Material

Supplemental Material

## Data Availability

The data that support the findings of this study are available from the corresponding author (Dragonkuilong@163.com) upon reasonable request.

## Highlights

miR-409 expression was downregulated in pancreatic carcinoma tissues and cells.

miR-409 was closely associated with lymph node metastasis, TNM staging.

Patients with low miR-409 expression in tissues exhibited a poor overall survival rate.

Upregulation of miR-409 may suppress tumor cellular behaviors by targeting GAB1.

miR-409 may be a potential prognosis candidate for the treatment of PC patients.
