# Supplementary material for: The clinical significance of microRNA-409 in pancreatic carcinoma and associated tumor cellular functions
Source: Bioengineered. 2021 Aug 2;12(1):4633–42. doi: 10.1080/21655979.2021.1956404 (PMC8806886; doi:10.1080/21655979.2021.1956404)
Supplement: Supplemental Material [file KBIE_A_1956404_SM2323.zip › Supplementary table 1.docx]

**Supplementary Table 1** The potential target gene of miR-409 in PC using TargetScan, miRDB, and CancerMIRNome.

| Target genes | Representative transcript | Gene name |
| --- | --- | --- |
| GAB1 | ENST00000262995.4 | GRB2-associated binding protein 1 |
| ATXN3 | ENST00000545170.1 | ataxin 3 |
| MTF2 | ENST00000370298.4 | metal response element binding transcription factor 2 |
| SEC62 | ENST00000337002.4 | SEC62 homolog (S. cerevisiae) |
| ELF2 | ENST00000394235.2 | E74-like factor 2 (ets domain transcription factor) |
| ZEB1 | ENST00000361642.5 | zinc finger E-box binding homeobox 1 |
| RDX | ENST00000343115.4 | radixin |
| GNAL | ENST00000334049.6 | guanine nucleotide binding protein (G protein), alpha activating activity polypeptide, olfactory type |
| NUFIP2 | ENST00000225388.4 | nuclear fragile X mental retardation protein interacting protein 2 |
| ZFHX4 | ENST00000521891.2 | zinc finger homeobox 4 |
